# Feedback-Based, System-Level Properties of Vertebrate-Microbial Interactions

**DOI:** 10.1371/journal.pone.0053984

**Published:** 2013-02-20

**Authors:** Ariel L. Rivas, Mark D. Jankowski, Renata Piccinini, Gabriel Leitner, Daniel Schwarz, Kevin L. Anderson, Jeanne M. Fair, Almira L. Hoogesteijn, Wilfried Wolter, Marcelo Chaffer, Shlomo Blum, Tom Were, Stephen N. Konah, Prakash Kempaiah, John M. Ong’echa, Ulrike S. Diesterbeck, Rachel Pilla, Claus-Peter Czerny, James B. Hittner, James M. Hyman, Douglas J. Perkins

**Affiliations:** 1 Center for Global Health, University of New Mexico, Albuquerque, New Mexico, United States of America; 2 Population Health and Pathobiology, North Carolina State University, Raleigh, North Carolina, United States of America; 3 Department of Zoology, University of Wisconsin-Madison, Madison, Wisconsin, United States of America; 4 Animal Pathology, Università degli Studi di Milano, Milan, Italy; 5 Kimron Veterinary Institute, Bet Dagan, Israel; 6 Animal Sciences Department, Microbiology and Animal Hygiene, Faculty of Agricultural Sciences, Georg-August-University, Göttingen, Germany; 7 Biosecurity and P. Health, Los Alamos National Laboratory, Los Alamos, New Mexico, United States of America; 8 Human Ecology, Centro de Investigación y de Estudios Avanzados (CINVESTAV), Mérida, Yucatán, México; 9 Regierungspräsidium Gießen, Wetzlar, Germany; 10 Department of Health Management, Atlantic Veterinary College, University of Prince Edward Island, Charlottetown, Canada; 11 Department of Psychology, College of Charleston, Charleston, South Carolina, United States of America; 12 Department of Mathematics, Tulane University, New Orleans, Louisiana, United States of America; Centro de Pesquisa Rene Rachou/Fundação Oswaldo Cruz (Fiocruz-Minas), Brazil

## Abstract

**Background:**

Improved characterization of infectious disease dynamics is required. To that end, three-dimensional (3D) data analysis of feedback-like processes may be considered.

**Methods:**

To detect infectious disease data patterns, a systems biology (SB) and evolutionary biology (EB) approach was evaluated, which utilizes leukocyte data structures designed to diminish data variability and enhance discrimination. Using data collected from one avian and two mammalian (human and bovine) species infected with viral, parasite, or bacterial agents (both sensitive and resistant to antimicrobials), four data structures were explored: (i) counts or percentages of a single leukocyte type, such as lymphocytes, neutrophils, or macrophages (the classic approach), and three levels of the SB/EB approach, which assessed (ii) 2D, (iii) 3D, and (iv) multi-dimensional (rotating 3D) host-microbial interactions.

**Results:**

In all studies, no classic data structure discriminated disease-positive (D+, or observations in which a microbe was isolated) from disease-negative (D–, or microbial-negative) groups: D+ and D– data distributions overlapped. In contrast, multi-dimensional analysis of indicators designed to possess desirable features, such as a single line of observations, displayed a continuous, circular data structure, whose abrupt inflections facilitated partitioning into subsets statistically significantly different from one another. In all studies, the 3D, SB/EB approach distinguished three (steady, positive, and negative) feedback phases, in which D– data characterized the steady state phase, and D+ data were found in the positive and negative phases. In humans, spatial patterns revealed false-negative observations and three malaria-positive data classes. In both humans and bovines, methicillin-resistant *Staphylococcus aureus* (MRSA) infections were discriminated from non-MRSA infections.

**Conclusions:**

More information can be extracted, from the same data, provided that data are structured, their 3D relationships are considered, and well-conserved (feedback-like) functions are estimated. Patterns emerging from such structures may distinguish well-conserved from recently developed host-microbial interactions. Applications include diagnosis, error detection, and modeling.

## Introduction

The rate of undetected infections remains markedly elevated and may be increasing [1]–[3].

Pathogens that develop resistance to antimicrobials pose new challenges, such as methicillin- or multidrug-resistant *Staphyloccocus aureus* (MRSA) infections which, in the USA, cause more deaths than tuberculosis, AIDS, and viral hepatitis combined [4]. Macro-parasite-mediated diseases are also associated with high levels of drug resistance [5]. To enhance the detection of infectious disease-related data patterns, new approaches are required.

To that end, systems biology (SB) and evolutionary biology (EB) may be considered. To diminish data variability, EB focuses on biological features well conserved in evolution [6]–[12]. However, in infectious diseases, EB has not yet provided usable methods [6]. Unlike reductionist approaches, which only consider a few and static variables, SB focuses on systems and their dynamics –a feature that may extract more information from the same data [13]–[18].

However, before SB/EB concepts are explored within the context of infectious diseases, we need to remind ourselves that we live in a three-dimensional (3D) environment [19]. And yet, the data we are exposed to are mainly ‘flat’, such as anything reported on a page or screen. Such formats are bi-dimensional: they lack the third dimension (depth). Bi-dimensional (2D) data formats are poor (if not also, biased) descriptions of three- (four- and/or multi-) dimensional data structures. Only 3D plots (volumes) can express all the combinations (points, lines, or surfaces) biological data can generate [20]. Furthermore, rotating 3D plots could inform whether perspective (the angle under which the data are assessed) influences pattern detection [21].

In spite of such possibilities, 3D data analysis seems to be under-utilized in the area of infectious diseases. In October of 2012, a search conducted in the Web of Science^©^ yielded >18,000 hits when ‘three-dimensional’ and ‘data analysis’ were queried, but less than 100 hits were retrieved when ‘infection’ was added.

While feedback is a function of interest in both SB and EB and it has been known for at least half a century in medicine and two millennia in physics [22]–[25], feedback has only marginally been explored in infections. In October of 2012, more than 200,000 bibliographic hits could be retrieved under ‘feedback’ and ∼1700 hits were yielded when ‘feedback’ and ‘definition’ were searched for, but less than 50 hits were found when ‘infection’ was added. Even though the precursor of feedback (‘homeostasis’) was first proposed in 1932 [26] and, in 1956, the phrase ‘negative feedback’ was first published in biology [27], only after the concept was introduced in engineering, feedback was fully adopted in biology. After the emergence of system dynamics, non-linear approaches have been applied to study feedback phases [28].

In its simplest version, *feedback* can be defined as the ability of a system to adjust its output in response to monitoring itself [29]. An expanded definition, which defines as dynamic any situation in which some quantity increases or decreases over time [30], [31], regards feedback as a process that involves an interaction between two or more elements (e.g., a microbe and a host), which is designated *positive* when the activation or accumulation of one component leads to the activation or accumulation of the other component, and *negative* when the activation or accumulation of one component leads to the deactivation or depletion of the other component [29]. *Positive* feedback occurs when a signal induces more of itself, or of another molecule that amplifies the initial signal, and this serves to stabilize, amplify or prolong signaling. *Negative* feedback occurs when a signal induces its own inhibition [29].

Feedback exhibits loops or closed chains in which change in one component is fed back to its origin [31], [32]. Other feedback structures are: (i) nodes, (ii) cyclic data patterns, (iii) directionality, and (iv) connectivity [24], [31], [33]. ‘Nodes’ refer to data groups where processes begin and end, and/or where data inflections may occur. Thus, feedback is a deterministic process, characterized by abrupt transitions from low to high (or high to low) activity [24], [31]. When high-level structures are assembled, feedback also reveals emergent properties [7]–[9].

Feedback emergent properties (the result of combinatorial theory and organizational complexity) can be explained with a mundane example that involves language. When we consider any list of letters, no meaning is obtained. However, when a few letters are combined, words emerge – and, with them, meaning emerges. When we combine words, sentences emerge, which elicit more information. Information does not depend on any one letter: it depends on combinations of letters (words). While low-level data (letters) lack information, information is created (and increases) when higher levels (words, sentences, paragraphs, and so forth) are used. Typically, rich (interpretable and usable) information emerges from the highest of such levels.

Similarly, the ability of a biological system to perform many functions with a few resources depends on its combinatorial potential, which is expressed as multiple structural levels [34]. Therefore, to design a method that discriminates infectious disease-related data patterns, at least three aspects or features should be considered: 1) multi-dimensionality, 2) combinatorial theory, and 3) various structural levels.

However, ‘level’ is an elusive concept. On the one hand, it may be synonymous with ‘organizational complexity’, which may be a dimensionless concept. On the other hand, ‘level’ may be measurable and synonymous with ‘scale’, as in the continuum that includes molecular, cellular, multi-cellular, organ, individual, population (group of individuals), species, groups of species (e.g., vertebrates), and ecological scales. Because both connotations may apply, new methods should adopt indicators inherently combinable, which are applicable across biological scales and can assess relationships, such as those created by multi-cellularity [35], [36].

Such relationships, to be detected, require ‘functional data integrity.’ By that we refer to the fact that the anti-microbial immune system is indivisible and, consequently, no leukocyte type ever works alone. ‘Functional data integrity’ alludes to the ability of measuring interactions (multi-factor relationships), not just one element [14]. Unlike ‘elementary variables’, ‘structured indicators’ can estimate functions, e.g., early anti-microbial responses.

The difference between ‘elementary variables’ and ‘structured indicators’ has been described before. While an ‘indicator’ possesses *links* – which establish a *temporal connectivity* and, therefore, reveal *directionality* and *causality* –, a simple variable, such as the percentage of neutrophils, lacks such information [31]. Hence, ‘functional data integrity’ summarizes all previous concepts with an observable set of properties: (i) it is the opposite of ‘fragmentation’– it includes data from all cells of the immune system, i.e., it possesses ‘integrity’, (ii) it is inherently combinatorial, that is, it may generate a large number of ‘words’ and, probably, ‘sentences’, even though its primary components (cell types) are as few as or fewer than the letters of any language; and (iii) such combinations may ultimately gauge critical biological functions, such as feedback functions – which may emerge from interactions that involve several biological scales and, to be optimally detected, should be measured in 3D space. That translates as measuring not the percentage of a single cell type but, for instance, the ratio between lymphocytes and macrophages – a multi-cellular interaction essential in antigen recognition [37].

To measure interactions, compositional data may be considered. Compositional data can provide relative information (information on one factor in relation to another). Such information is based on the use of ratios [38], [39]. Leukocyte data are compositional: their relationships can be expressed as relative ratios [40]. Compositional data possess scale invariance: informative (interpretable and usable) data patterns can be expressed, regardless of the (molecular/cellular/ multi-cellular/organ/population/species/ecological) scale of the data [41]–[43].

To complete the list of desirable criteria an informative method should possess, data variability (‘noise’) should be reduced and pattern recognition should be enhanced. Noise is reduced, if not eliminated, when a single line of observations is generated. Data patterns, if present, are likely to be detected when a single line of data points is observed.

Informative patterns, such as data inflections, as well as a single line of data points, can be generated when these conditions are met: (i) functional data integrity is applied (data from all cell types are considered), (ii) a 2D plot is created in which, on one axis, the percentage of one cell type is expressed, and a ratio is recorded on the second axis, and (iii) the denominator of such ratio is the same percentage expressed on the first axis. We call such indicators ‘anchors’, e.g., the 2D set that includes the lymphocyte (L) % (axis 1) and the phagocyte (macrophage [M] and neutrophil [N])/L ratio (axis 2). Because, in this structure, all data points are ‘anchored’ along a single line, noise is substantially reduced. Because, to build ‘anchors’, only two axes are required, a third axis remains available, in a 3D plot, to assess any additional variable.

Discrimination is also improved when bio-numerical properties are considered in the design of the indicators, as when two ratios are plotted together, and the numerator of one ratio is the denominator of the other ratio (e.g., the neutrophil per lymphocyte ratio [N/L ratio] vs. the mononuclear cell [MC, or L and M]/N [MC/N ratio]). In such a structure, when one ratio increases, the other ratio decreases. This structure acts as an ‘amplifier’: even when changes are quantitatively small, distinct (usually orthogonal) patterns can be revealed.

When ‘amplifiers’ are used and biological knowledge is included in the design, temporal changes can be assessed. That can be achieved when one ratio estimates early host-microbial responses and the other ratio expresses late responses. For example, a 2D plot that includes N/L and MC/N ratios indicates early responses when the N/L ratio is high (e.g., much greater than 1), or late responses when the MC/N ratio is >1 [44]–[46]. Such structure can distinguish the temporal sequence of biological responses regardless of chronological scales (minutes/hours/days) and is robust to the absence (or presence) of slow (or fast) immune responders [47].

While the cyclic nature of feedback features is useful to describe dynamics [48]–[50], to detect infectious disease dynamics, logical aspects should also be addressed. Fallacies may occur at the earliest stage of an investigation, when a hypothesis is postulated. For instance, when the hypothesis assumes that only two alternatives are possible (e.g., one disease-positive [D+] and one disease-negative [D–] data class [51]), but three or more alternatives exist, errors will follow.

Hence, using assumption-free, structured indicators (designed to reduce noise and possess functional data integrity), the multi-dimensional patterns of host-microbial interactions were explored. Two questions were asked: 1) can SB/EB indicators reveal feedback phases? and 2) can such indicators be used to enhance the detection of infectious disease-related data patterns?

## Materials and Methods

### Materials

Leukocyte data and microbial test results were collected in: 1) bacterial infections induced by methicillin- or multidrug-resistant *Staphylococcus aureus* (MRSA) and non-MRSA bacterial infections of bovines and humans, 2) parasite (*Plasmodium falciparum*) infections that affected humans; and 3) viral (West Nile virus) infections experimentally induced in chickens. Six evaluations – three longitudinal and three cross-sectional studies – were conducted.

### Method

**Figure 1:**
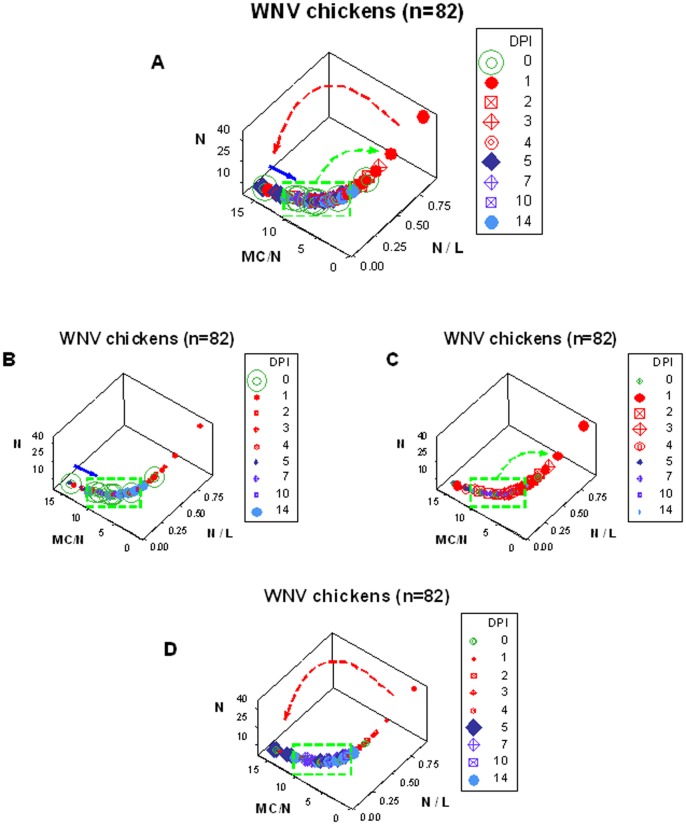
Feedback patterns of avian longitudinal-experimental immune responses against West Nile Virus. **A**: Leukocyte and microbial test results of 10 chickens (shown to be West Nile virus [WNV] negative at day 0) were inoculated with WNV and followed over two weeks (total: 82 longitudinal observations). The 3D relationship that included the heterophil (N) %, the ratio of N per lymphocyte (N/L), and the mononuclear cell/N (macrophage plus lymphocyte/N or MC/N) ratio showed three major data inflections: 1) a double 90-degree inflection was observed between pre-inoculation (0 dpi) and one day post-inoculation (1 dpi) data points (green arrow), indicating that the N/L ratio increased within a 24-hour period, and that high N/L observations were D+ (red symbols); 2) at, approximately, 5 dpi, a second data inflection was observed (red arrow), which was associated with high MC/N and low N/L values; and 3) soon after 5 dpi, the third data inflection took place, indicating the beginning of the return to the steady state phase (blue arrow). The third phase was characterized by the gradual decrease of MC/N values (deep blue symbols). The last phase ended when 14-dpi observations (sky blue symbols) displayed leukocyte values similar to those of 0-dpi (D–) data (green symbols, of which 80% were within the data range indicated by the green box). Together, a quasi-circular, closed, temporal progression was detected, in which three feedback phases were differentiated: 1) the steady state phase (green symbols), 2) the positive phase (red symbols), and 3) the negative phase (blue symbols). Because observations that differed less than 24 hours (0- vs. 1-dpi data) were clearly separated, these patterns could detect early inflammatory responses, even in the absence of microbial data. These patterns distinguished two D+ classes (red and blue symbols). Because D+ observations that revealed ‘overshooting’ (higher MC/N values than those of D– data) later approached the D– stage, D+ individuals showing high MC/N values may have a favorable prognosis. **B–D**: To facilitate visual detection of patterns specific of each feedback phase, the same data displayed in A are shown emphasizing: only the feedback steady state phase (**B**), only the early (positive feedback) phase (**C**), and only the late (negative feedback) phase (**D**). Utilizing a different quantitative method, these data have been partially reported elsewhere [52].

Leukocyte data (heterophils, granulocytes, or neutrophils [N]; macrophages or monocytes [M]; and lymphocytes [L]) were structured as described earlier. Leukocyte and microbial procedures are described in Text S1 of Supporting Information, which includes a glossary [52]-[64]. Briefly, tables, generic, and goal-related analyses were created or processed as follows:

1. Data organization and table building.
  - A table was created in which columns included primary variables (L%, N%, M%, their counts, as well as microbial test results).
  - Additional columns included secondary variables, e.g., the percentages of (a) phagocytes (P, or N+M), (b) mononuclear cells (MC, or L+M), and (c) the remaining alternative, here named ‘small leukocyte’ (SL, or L+N).
  - Later, tertiary variables were added to new columns, which denoted interactions, such as the N/L, M/L, M/N, P/L, MC/N, and SL/M ratios; e. g., the N/L ratio was calculated by dividing the N% over the L%. Hence, 12 leukocyte-related variables were created from the 3 original percentages (through combinations, the number of variables was expanded four times). However, more combinations were generated when the analysis was conducted.
2. Generic analysis.
  - When the goal was to produce a single line of data points, ‘anchors’ were selected.
  - When enhanced discrimination was pursued, ‘amplifiers’ were chosen; for instance, if a 2D plot was used, the N/L ratio was plotted on one axis and the M/N ratio on the other.
  - When both effects were pursued, a 3D plot was utilized and one variable performed two roles, e. g., the set that includes the N%, the MC/N and N/L ratios is both an ‘anchor’ (MC/N vs. N%) and an ‘amplifier’ (the N/L vs. the MC/N).
  - Because a ratio has two expressions (such as the L/M and the M/L ratios), both versions of each ratio were analyzed (a strategy that doubled the number of possible analyses).
3. Applications or goal-oriented analysis.
    - To enhance discrimination, 3D plots were rotated until a data inflection was displayed and one corner of the plot displayed the zero value of all the three axes, as shown in Figure 1.
    - To determine directionality, temporal data were assessed.
    - To detect emergent properties, microbial data were considered.
    - To investigate the role of perspective, 3D plots were rotated.
    - To explore robustness, different species/pathogens were analyzed under the same angle.
    - To distinguish different subsets of the same data class, the 3D plot was rotated until the highest values of two indicators were observed on opposite corners (as shown in Figure 1). In addition, the size of symbols representing non-relevant features was decreased, so only the features of interest were emphasized, e.g., if the goal was to detect false negatives, D+ symbols were reduced; if the goal was to identify ≥2 D+ stages, D– symbols were reduced.

## Results

### Feedback-related patterns

The use of SB/EB indicators in an experimental study of virally-infected chickens revealed 10 properties or features: 1) functional data integrity, 2) a single line of data points, 3) data inflections, 4) a circular data structure, 5) directionality of the temporal responses, 6) patterns that suggested three feedback phases, 7) two distinct D+ subsets, 8) overshooting (a D+ subset with higher MC/N values than D– observations), 9) information of prognostic value, and 10) low data variability (Figure 1 A).

*Functional data integrity* was achieved because each observation expressed values contributed by all cell types. Each data point estimated three interactions: 1) the relationship between neutrophils and lymphocytes, 2) that between mononuclear cells and neutrophils, and 3) the overall or ‘high-level’ interaction, generated by the two interactions mentioned above.

The observed *single line of observations* revealed *circularity*, which was characterized by three major *data inflections*: the first inflection was observed within one day post-inoculation (1 dpi) with West Nile virus (WNV, green arrow, Figure 1 A); around 5 days post-inoculation (5 dpi), a second data inflection was observed (red arrow, Figure 1 A); which was followed, almost immediately, by the last inflection (blue arrow, Figure 1 A). Because temporal observations displayed *directionality*, three data ranges were distinguished in Figure 1 A: 1) that of the *steady feedback* phase (0 dpi or D– data [green symbols, of which 80% were within the range indicated by the green box]); 2) away from the steady state phase (between 1 and 5 dpi), in which D+ data predominated (*positive feedback* phase [red symbols]); and 3) the *negative feedback* phase (after 5 dpi), in which, over time, D+ data (blue symbols) approached the data range of the steady state phase. The end of the feedback function was signaled when the latest (14-dpi) observations reached values similar to those they started with (sky blue symbols).

Hence, *two D+ subsets* (early D+ and late D+ observations) were distinguished. The late D+ subset was characterized by high MC/N values –observed around 5 dpi–, which displayed *overshooting*, that is, greater MC/N values than those of D– data points. Because the latest (14- dpi) D+ data points did not differ from D– values, it was concluded that high MC/N, D+ individuals had a favorable *prognosis*: such pattern indicated the beginning of the return to the steady state phase. Because most early and late observations were located on opposite sides of the plot analyzed, both *low variability* and *enhanced discrimination* were documented (Figure 1 A). To facilitate visualization, each feedback phase is emphasized in Figures 1B–D.

### Reproducibility of feedback-like patterns

**Figure 2:**
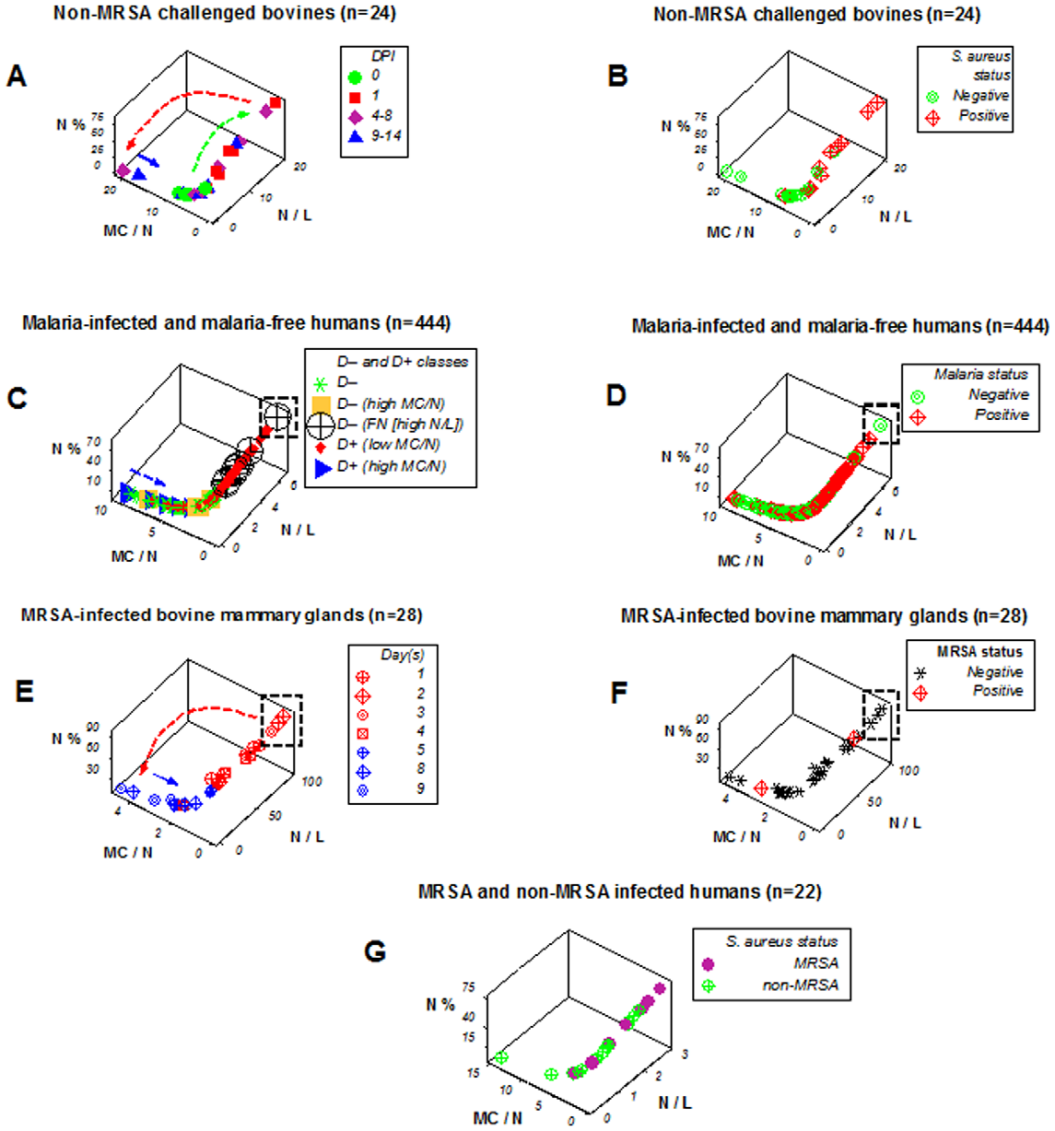
Reproducibility of feedback patterns across species and pathogen types. Bovines and humans exposed to either bacteria (both sensitive and resistant to anti-microbials) or parasites showed patterns similar to those displayed by birds (**A**–**F**). **A**, **B**: Leukocyte profiles and microbial test results of 6 dairy cows inoculated at day 0 with non-methicillin resistant (non-MRSA) *Staphylococcus aureus*, followed over two weeks, are reported (total: 24 longitudinal observations, data previously reported, using a different analytical method [44]). **C**, **D**: Leukocyte profiles of 439 humans non-infected or infected by malaria, tested once, are reported, of whom five displayed high MC/N values and malaria-positive test results and were tested twice (two weeks apart), becoming malaria-negative in the later test (total: 444 observations, data previously reported, using a different analytical method [61]). **E**, **F**: Longitudinal profiles of bovine mammary gland leukocytes collected from a cow spontaneously infected with methicillin-resistant *S. aureus* (MRSA) are shown (total: 28 longitudinal observations or 7 daily tests per mammary gland, a study previously reported, in which a different analytical method, a different technology, and different samples were utilized [54]). **G**: Cross-sectional leukocyte profiles of humans infected by either MRSA (n = 7) or non-MRSA (n = 15) bacterial isolates are described (data not previously reported). DPI: day(s) postinoculation with non-MRSA. DAYS: consecutive days since MRSA was isolated (day 1 = day of first isolation). Left columns show temporal data, in longitudinal studies (**A**, **E**); or disease-positive (D+) and disease-negative (D–) malaria-related data subsets (**C**). Right columns display microbial test results (**B**, **D**, **F**, **G**). Microbial-negative results that revealed high N/L values were suspected to be false negative (boxes, **C**–**F**). In the malaria study, 8 false negatives were detected (8 black circular symbols, of which one is shown within a box, **C**), which were associated with fever. Arrows indicate the directionality of temporal responses (**A**, **C**, **E**). **H**: To facilitate visual detection of patterns, the same data displayed in plot C are shown again, with emphasis on D– data (the symbols of D+ data are reduced in size). A data inflection is observed, which distinguishes two D– subsets. The high N/L subset (black polygon, **H**), as indicated in the main text, was suspected (and later confirmed) to include false negatives.

The reproducibility of feedback patterns was investigated across species and diseases (Figures 2 A–H). Longitudinal bovine leukocyte profiles, assessed together with bacteriological test results (Figures 2 A, B), showed patterns similar to those observed in birds, such as a single line of data points, circularity and directionality (arrows, Figure 2 A). While the, predominantly, cross-sectional nature of the human data prevented the full determination of temporal features (Figures 2 C, D), a subset of 5 D+ children, who were tested twice, also revealed, partially, the directionality shown by birds and cows: a group of high MC/N, D+ children, when tested two weeks later, was D– (blue arrow, Figure 2 C). The fact that 5 children (D+ at their first test) were D– two weeks later, suggested, again, that leukocyte-microbial profiles (high MC/N, D+ data) can have prognostic applications. Even though the spontaneous nature of bovine MRSA infections could not show a D– profile (no ‘day 0’ data were available, Figures 2 E, F), the bovine MRSA data revealed the same (early vs. late) temporal patterns observed in other studies (arrows, Figure 2 E). Although longitudinal data were not available in the study in which humans were infected by bacteria, a distinct pattern was observed: MRSA observations did not express overshooting (no MRSA infection displayed high MC/N values), while non-MRSA data did (Figure 2 G). False negative results were suggested by human and bovine data profiles: some hHHigh N/L values were associated with microbial-negative results (black boxes, Figures 2 C–F). The false negative hypothesis was confirmed in humans: 8 microbial-test negative, high N/L children were febrile (8 black circular symbols, one within a black box, Figures 2 C, D, H).

### From no discrimination to discrimination of host-microbial interactions

**Figure 3:**
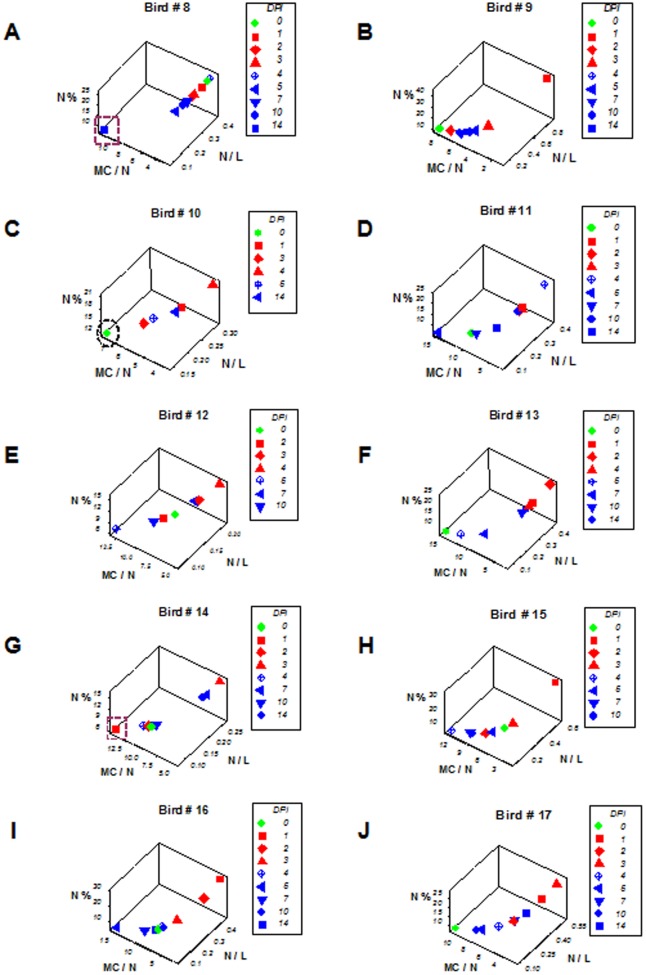
Responsiveness of individuals – avian examples. The same avian data previously analyzed at the population scale (Figures 1 **A–D**) were assessed at the individual scale (**A-J**). Even though chickens were selected through randomization, high variability was found. For instance, two birds (# 8 and 14) were fast responders: as early as 1 day-post inoculation (dpi) with West Nile Virus, they showed leukocyte profiles typical of the late or negative feedback phase (square boxes, **A, G**), In contrast, at least one bird did not display overshooting (no D+ observation of bird #10 displayed MC/N values greater than the D– [0-dpi] data point, oval, **C**).

Discrimination of health status was lost when individuals, not populations, were analyzed (Figures 3 A–J). Some birds were fast responders –they showed patterns typical of late D+ responses as early as one day after challenge (boxes, Figures 3 A, G), while one bird did not display high MC/N values at any time (oval, Figure 3 C). Such differences in responsiveness were observed even though the birds included in this study were randomly selected.

**Figure 4:**
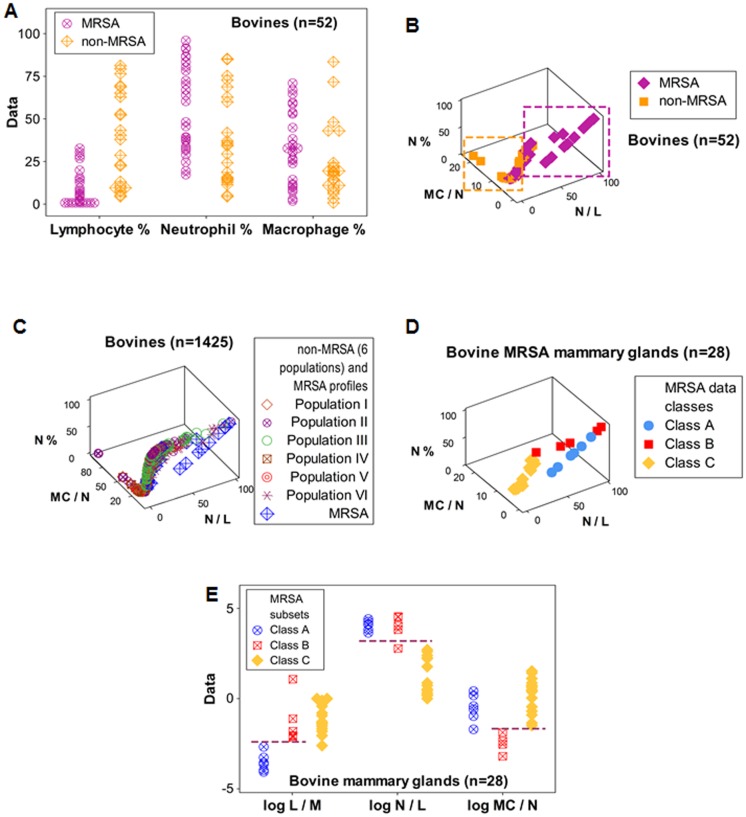
Discrimination between bovine MRSA and non-MRSA patterns. While no leukocyte percentage discriminated between methicillin- or multidrug-resistant *S. aureus*–infected (MRSA) and non-MRSA bovines (**A**), a three-dimensional (3D) plot that utilized SB/EB indicators distinguished MRSA from non-MRSA patterns, e. g., MRSA observations displayed higher N/L values than non-MRSA data points, while higher MC/N values were revealed by non-MRSA observations (**B**). The MRSA profile was differentiated even when compared against a large, cross-sectional bovine dataset (**C**). Regardless of microbial test results, 3 MRSA data classes were detected (**D**). When, based on 3D patterns, the MRSA data were partitioned, each MRSA data class (A, B, C) was distinguished by one or more indicators, and non-overlapping distributions were observed, which differed from one another at statistically significant levels (*P*<0.01, Mann-Whitney test, Table 1, **E**). Horizontal lines indicate full discrimination (non-overlapping data distributions) between two or more MRSA classes (**E**). Although utilizing a different quantitative method, bovine cross-sectional data of populations II-VI (**C**) have been partially or totally reported before [55]–[59].

Discrimination was also lost when ‘functional data integrity’ was not considered (when each leukocyte type was assessed alone). When only the percentage of neutrophils (lymphocytes, or macrophages) was assessed, bovine MRSA and non-MRSA data overlapped (Figure 4 A).

In contrast, SB/EB indicators distinguished non-MRSA from MRSA data: while non-MRSA data displayed ‘left overshooting’ –higher MC/N values than those of MRSA data points–, all four mammary glands of the MRSA cow showed ‘right overshooting’ (higher N/L values than those of non-MRSA infections, Figure 4 B). The MRSA profile was detected even when compared against a large, cross-sectional, non-MRSA dataset (Figure 4 C, Table 1; see Table 2 for further details on the non-MRSA, cross-sectional data). Even though no MRSA was isolated in three bovine mammary glands, all four mammary glands of the MRSA cow showed similar leukocyte profiles, which revealed 3 data classes (A, B, and C). Figure 4 D shows that the distributions of classes A–C did not overlap. All three MRSA data subsets were statistically differentiated by, at least, one indicator (*P*<0.01, Mann-Whitney test, Table 1; and Figure 4 E).

**Table 1:** Comparisons between MRSA and non-MRSA profiles, and among MRSA subsets.

Data classes or subsets	Variables	*P* value (Mann-Whitney test)
Non-MRSA (all observations) vs. MRSA	N/L	<0.01
Non-MRSA (all observations) vs. MRSA	MC/N	<0.01
Non-MRSA post-challenge vs. MRSA	N/L	<0.01
Non-MRSA post-challenge vs. MRSA	P/L	<0.01
MRSA class A vs. MRSA class B	SL/M	<0.01
MRSA class A vs. MRSA class B	M/N	<0.01
MRSA class A vs. MRSA class B	P/L	<0.01
MRSA class A vs. MRSA class C	N/L	<0.01
MRSA class A vs. MRSA class C	P/L	<0.01
MRSA class B vs. MRSA class C	N/L	<0.01
MRSA class B vs. MRSA class C	MC/N	<0.01

**Table 2:** Cross-sectional bovine non-MRSA infections.

Population	Prevalence (%)		Examples of bacterial species isolated for studies I-VI	
	Major pathogens	Minor pathogens	Major pathogens	Minor pathogens
CS I (n = 120)	27.5%	13.3%	*Staphylococcus aureus; Escherichia coli; Streptococcus dysgalactiae; Streptococcus uberis; Klebsiella pneumoniae*	*Staphylococcus chromogenes;* *Staphylococcus hyicus; Corynebacterium* sp
CS II (n = 500)^1^	9.8%	5.2%		
CS III (n = 429)^2^	9.9%	5.3%		
CS IV (n = 80)^3^	2.5%	2.5%		
CS V (n = 80)^4^	6.5%	23.8%		
CS VI (n = 188)^5^	13.3%	11.2%		

1 . Raw data partially or totally reported elsewhere [55].

2 . Raw data partially or totally reported elsewhere [56].

3 . Raw data partially or totally reported elsewhere [57].

4 . Raw data partially or totally reported elsewhere [58].

5 . Raw data partially or totally reported elsewhere [59].

**Figure 5:**
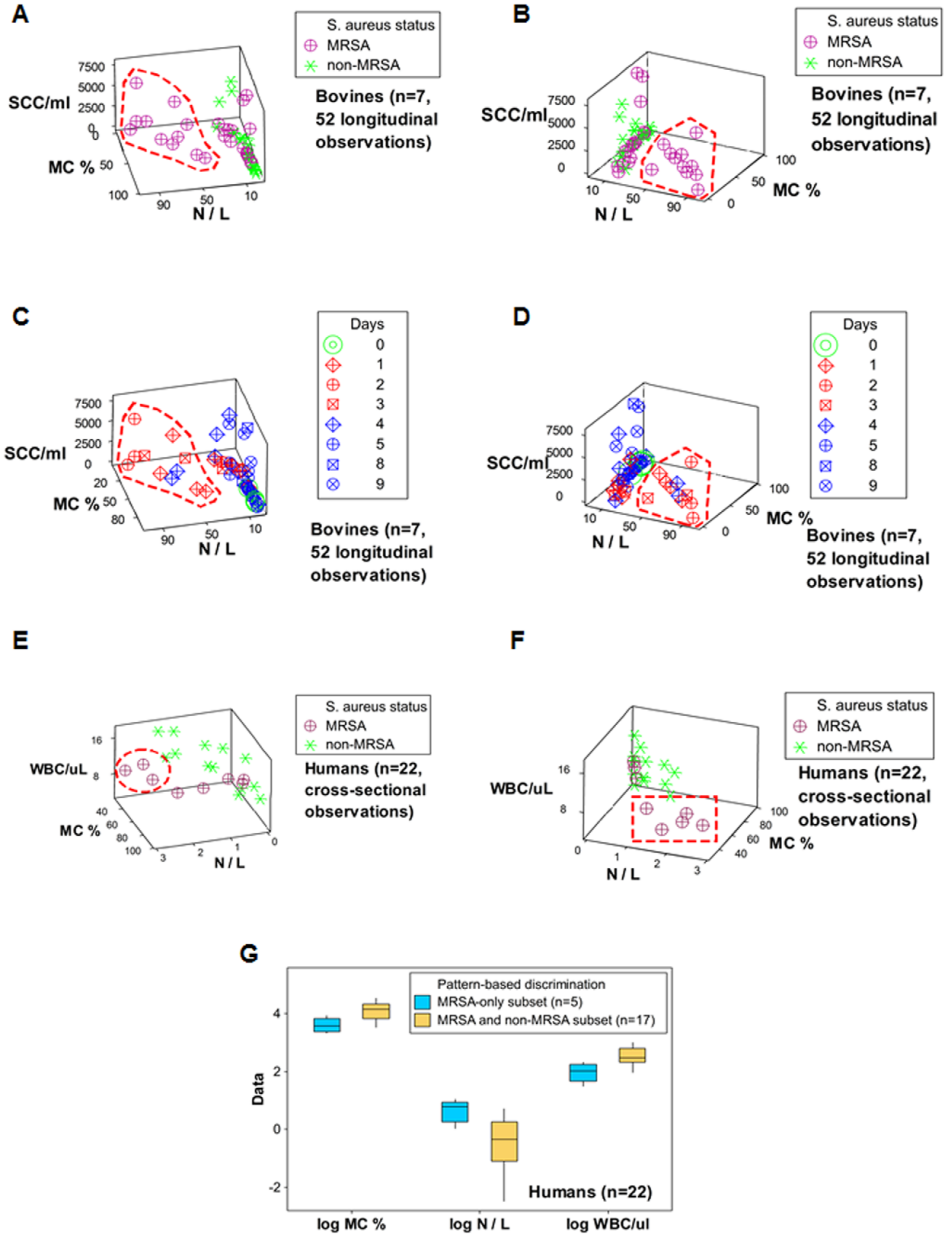
The role of perspective: discrimination between early MRSA and other (MRSA and non-MRSA) patterns, in bovines and humans. MRSA and non-MRSA induced leukocyte profiles were investigated in bovines and humans. In both species, non-MRSA individuals were infected by methicillin-susceptible *S aureus*. Two 3D perspectives of the same data were analyzed in MRSA and non-MRSA bovine infections (**A, B**). When the total leukocyte count (thousands of milk cells or ‘somatic cell counts’ [SCC]) was assessed together with the mononuclear cell (MC) percent and the N/L ratio, two data subsets were differentiated: one was characterized by MRSA-only observations (red polygon), while the other data subset included both MRSA and non-MRSA observations (**A, B**). The MRSA-only subset was predominantly composed of early observations (days 1–4, red polygon, **C, D**). When human blood leukocyte counts (hundreds of white blood cell [WBC] counts), collected from MRSA and non-MRSA infected individuals were investigated, a MRSA-only subset was observed, which was defined by the same parameters utilized with the bovine data: low MC% and high N/L values (red circle or rectangle, **E, F**). Because the number of observations found within the subset that only included MRSA observations ranged between three (**E**) and five data points (**F**), it was demonstrated that the angle under which the data are analyzed is relevant: if perspective is considered, greater discrimination may be achieved. Three leukocyte indicators distinguished the two human (MRSA-only vs. MRSA and non-MRSA) subsets (*P*<0.01, Mann-Whitney test, **G**).

To determine whether perspective influences pattern detection, both human and bovine *S. aureus*-positive data were analyzed under different angles. The set that included leukocyte counts (human white blood cells or WBC, and bovine somatic cells or SCC [milk cells mainly composed of leukocytes]), the percentage of mononuclear cells (MC %) and the N/L ratio revealed a subset of data points that was only or mainly composed by MRSA observations (red polygons or circles, Figures 5 A–F). This subset did not overlap with the remaining (MRSA and non-MRSA) data points. When the data were analyzed under two different angles, between three and five data points were found within the human MRSA-only cluster (Figures 5 E, F). Hence, perspective may indeed alter the number of observations detected with a particular feature.

Because the human and bovine MRSA-only clusters revealed similar values (Figures 5 C-F) and the bovine cluster included the earliest observations (days 1–4, Figures 5 C, D), the MRSA-only cluster was suspected to express early infections. The early (MRSA-only) cluster differed statistically from the remaining data points (*P*<0.01, Mann-Whitney test, Figure 5 G).

**Figure 6:**
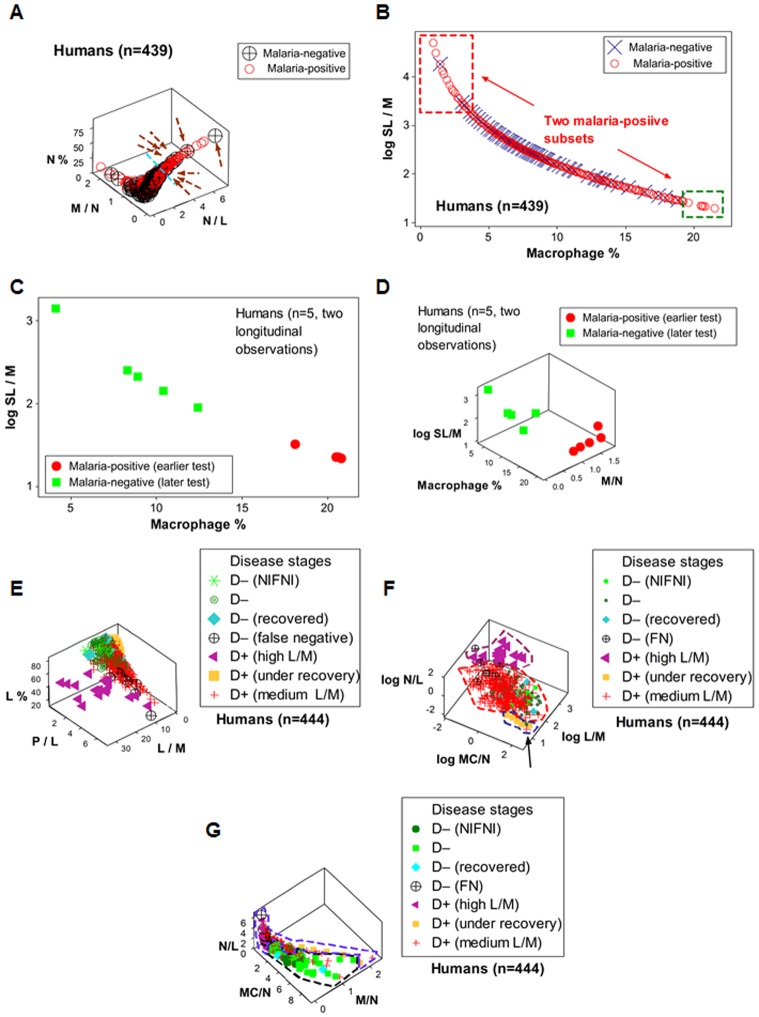
Detection of false negatives, prognosis, and three D+ data subsets in human malaria. The process by which false negative results were assessed is illustrated with data collected from humans infected or not infected by malaria. Arrows indicate 8 parasite-negative results, which were associated with high N/L values (**A,** also shown in Figures 2 C, D, H). Clinical data corresponding to the 8 children revealed that all of them were febrile. Spatial data patterns facilitated the detection of the 8 false negative (FN) results: an orthogonal data inflection (sky blue line) separated the data range in which the 8 FN points were found from the area in which D+ data predominated (**A**). Other spatial data patterns identified a subset associated with a favorable prognosis: two D+ subsets (arrows, **B**) were separated from one another by a segment in which both D+ and D– data points were observed, suggesting that the two D+ subsets observed at both ends of the plot could differ functionally (boxes, **B**). The subset with the higher M% was suspected to be under recovery. When the 5 individuals within the high M% subset (red symbols, **C**) were tested again, two weeks later, all of them were D– (green symbols, **C**). The change in health status, which took place within two weeks, revealed an orthogonal 3D data inflection (**D**). An additional set of indicators (the L%, P/L and L/M ratios) detected a third D+ subset, which showed high L/M values and differed from all other subsets (purple triangles vs. other symbols, **E**). Based on spatial patterns (shown in Figure 2 and here), the data were partitioned into subsets, which differed from one another at statistical significant levels (all comparisons with *P*<0.03, Mann-Whitney test). The degree of non-overlapping data distributions between two or more subsets (discrimination) was 1/336 (arrow indicates the overlapping point, **F**) when the 3 D+ stages (characterized by high MC/N or under recovery [n = 5], medium L/M [n = 314], or high L/M [n = 17]) were assessed. Total discrimination (no overlapping or 0/130) was seen when 3 D+ data classes (under recovery [n = 5], high L/M [n = 17], and FN [n = 8]) were assessed vs. the 3 D– classes [n = 100]) and the set that included the N/L, MC/N, and M/N ratios was utilized (**G**).

Other indicators (that possessed functional data integrity but did not meet ‘anchoring’ criteria) confirmed the patterns shown by the indicators described above. For instance, in the malaria study, the indicator set that measured the M/N (not the MC/N) ratio identified the same 8 data points regarded to be false negatives (arrows, Figure 6A; data also shown in Figure 2 H).

When a different ‘anchor’ (composed of the SL/M ratio and the M%) was used to analyze the malaria data, two D+ subsets were distinguished (Figure 6 B). Because the D+ subset with the highest M % and lowest SL/M values indicated a recovery profile, children in that subset were examined 14 days later. At that time point, all previously D+ children were D– and showed a distinct, non-overlapping leukocyte profile (Figure 6 C). The changing pattern observed over two weeks, which supported a favorable prognosis, displayed a 3D data inflection (Figure 6 D).

A third D+ subset was found in the malaria data with a ‘hybrid’ set that included both ‘anchor’ (the P/L ratio vs. the L%) and ‘amplifier’ features (the P/L and L/M ratios, Figure 6 E). Such structure facilitated data partitioning into subsets. Statistically significant differences were found: 1) between FN and all D– observations, 2) between every D+ subset and every D– subset, and 3) among the 3 D+ subsets (*P*≤0.03, Mann-Whitney test, Table 3).

**Table 3:** Differentiation of malaria classes.

Data classes	D– (n = 83)	D– NIFNI (n = 12)	D– (recovered, n = 5)	False D–(febrile, n = 8)	D+ high MCN/N (n = 5)	D+ low SL/M (n = 314)
D– NIFNI (n = 12)	NS					
D– recovered (n = 5)	NS	NS				
False D– (febrile, n = 8)	<0.01	<0.01	<0.01			
D+ high MC/N (n = 5)	<0.01	<0.01	<0.01*	<0.01		
D+ medium L/M (n = 314)	<0.01	<0.01	<0.03#	<0.01	<0.01	
D+ high L/M) (n = 17)	<0.01	<0.01	<0.01	<0.01*	<0.01	<0.01

The statistical results of human data reported in Figures 6 E-G (n = 444) are shown, where pairs of data classes are compared. The *P* values of analysis of medians (Mann-Whitney test) were determined by the MC/N ratio, the SL/M ratio (*), or the P/L ratio (#). NS: not significant at *P* = 0.05. The D– NIFNI group (neither infected, febrile, nor inflamed) is not a separate class, it is a reference for the overall D– class. See legend of Figures 6 E-G for further details.

Because statistical significance may be found even in the absence of discrimination (D+ and D– data overlapping may occur, even when median D+ and D– values differ statistically), the SB/EB approach was also assessed spatially. No data overlapping was found among: 1) the three D+ subsets (Figure 6 F); and 2) all three D– and two D+ (and FN) stages (Figure 6 G). The overlapping rate (percentage of observations assigned to one disease stage which showed values typical of another disease class) ranged between 0 (Figure 6 G) and 0.002 (1/336, one medium L/M D+ data point was found within the range of 336 high MC/N D+ data points, arrow, Figure 6 F). While spatial patterns did not distinguish some D+ (low or medium L/M) data points from D– data, such classes were differentiated on the basis of parasite test results (Figures 6 F, G).

### Assessment of percentages, ratios, counts, and hypothesis-related assumptions

**Figure 7:**
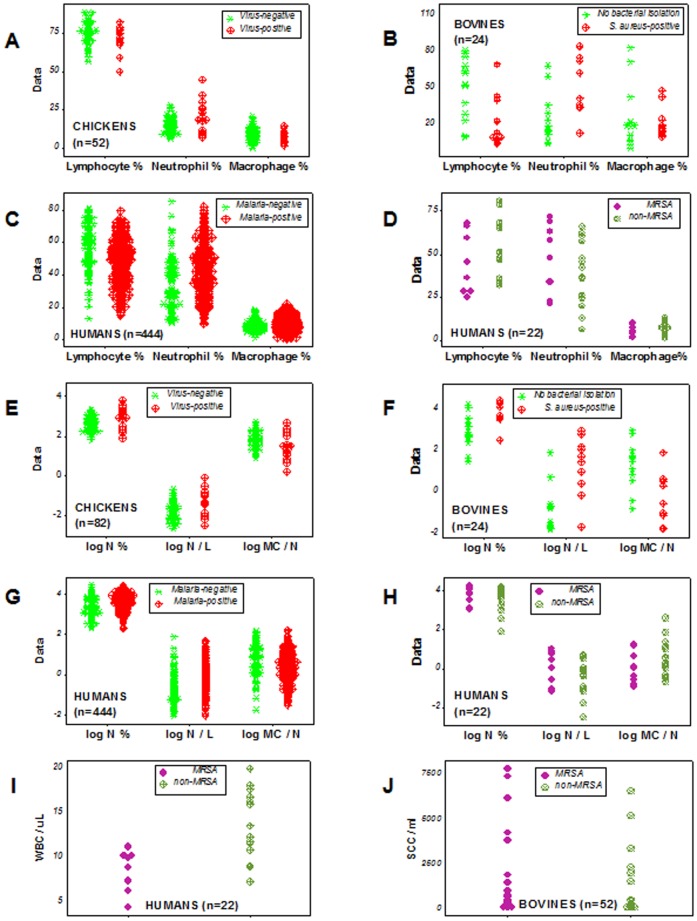
Assessment of ratios, counts, percentages, and hypothesis-related **assumptions.** When the SB/EB approach was not applied, the percentage of lymphocytes, neutrophils, or macrophages did not distinguish, in any study, D– from D+ data (**A–D**). Indicators that, together, detected patterns (the N%, the N/L and MC/N ratios), did not discriminate D– from D+ data when assessed individually (**E–H**). Total leukocyte counts also failed to distinguish health status: neither the human white blood cell count (WBC) nor the bovine milk total cell count (‘somatic cell count’ or SCC) differentiated D– from D+ data (**I, J**). Hence, findings supported several Systems Biology principles: 1) data integrity is necessary (because the immune system is indivisible, discrimination is lost when any leukocyte type is measured alone, **A–D**), 2) the format utilized is relevant: to detect ‘high-level’ interactions (those involving at least two interactions), 2D or 3D plots are required (as Figures 1, 2, 3, 4, 5, 6 show); and 3) emergence was demonstrated: while, individually, no indicator distinguished D– from D+ data (**A–H**), when 3D structures were assembled, D– and D+ data were distinguished (as shown, for instance, in Figure 2 H). Findings also demonstrated that statistical significance is not synonymous with discrimination: the median WBC count of human MRSA infections differed from the median WBC count of non-MRSA individuals (*P*<0.03, Mann-Whitney test), even though D– and D+ data overlapping was observed (**I**). However, when the data were structured as SB/EB indicators, both statistical significance and discrimination were achieved (as shown, for instance, in Figures 4, 5, 6). SCC: somatic cell counts (thousands)/ml. WBC: white blood cells (hundreds)/μl.

When SB/EB concepts were not applied, neither the L%, the N%, nor the M%, alone, differentiated, in any study conducted, D+ from D– data (Figures 7 A–D). When SB/EB concepts were not applied, neither log-transformations nor ratios distinguished data classes (Figures 7 E–H). In two species, cell counts did not distinguish MRSA from non-MRSA subjects (Figures 7 I, J). Hence, without the SB/EB approach, no primary variable, per se, could discriminate.

The validity of the ‘gold standard’ (the assumption that there is an ideal microbial test) was not supported in the human study on malaria: 8 children regarded as D– by the tests used were febrile (false negatives or FN, Figure 2 C). In the bovine MRSA study, only two out of 7 tests (performed with milk collected from the same mammary gland) yielded MRSA, that is, the ‘gold standard’ hypothesis failed 5 out of 7 times (a 71.3% false negative rate, see Figures 2 E, F). In contrast, in humans, SB/EB spatial patterns identified data points suspected to be FN: they were spatially distant from D– observations (Figures 2 C, H; and 6 E).

## Discussion

### Major findings

The SB/EB approach revealed similarities across vertebrate species (e.g., data circularity, Figures 1 and 2). Such approach also demonstrated differences within the same species and disease. For instance, high L/M values distinguished one malaria-positive subset [65] from other D+ subsets (Figure 6 E). Findings rejected: 1) the ‘gold standard’ hypothesis; 2) the binary hypothesis (only two, one D+ and one D–, data classes); and 3) the hypothesis that postulates randomization reduces variability. To interpret the findings, biological, statistical and methodological aspects are considered and their influence on theory is outlined.

### Biological and statistical considerations

In agreement with the theory that predicates the immune system is indivisible [66], no cell type, alone, discriminated D+ from D– subsets (Figure 7). It was also confirmed that dichotomizing approaches (which attempt to convert data inherently continuous into discontinuous data classes) are associated with D+ and D– data overlapping [67].

In contrast, discrimination was enhanced when interactions among all leukocytes were explored in a 3D space. Such approach measured or revealed *hierarchy*, *feedback, and emergence* [66]. ‘*Hierarchy*’ was assessed by focusing on the trans-vertebrate species set that also included several pathogen types. Such system revealed *feedback* loops. ‘*Emergence*’ was not revealed by any one primary component. Emergent properties, such as false negative patterns, were only detected when several levels of the biological system were assembled.

While SB/EB properties have been regarded to reveal low variability [12], avian data seemed to contradict such expectation. In spite of randomization [68], high data variability was shown by the fact that both low and fast responders were observed (Figure 3). High variability co-existed with low variability, as Figure 1 reveals.

To explain such an apparent contradiction, we could pose the following question: ‘how old are you: 44 million years old, or four years old?’ The answer is not ‘neither’ but, probably, ‘both.’ All vertebrates are ‘44 million years old’ because many of their critical structures are that old, if not older, such as mitochondria and the complement system [69]–[71]. Yet, a particular species (and an individual of a particular species) is much ‘younger’, e.g., the first chickens (*Gallus domesticus*) and hominids emerged in the last 3.6 million years [72], [73]. That means that individuals express biological functions that precede their own species and their own birth.

On the other hand, because the responsiveness of an individual can be shaped by unique experiences and pathogens can undergo mutations (such as MRSA), ‘new’ situations may arise. Because methicillin was introduced in 1960 [4], MRSA infections are recent evolutionary phenomena. Because vertebrates have not yet had enough time to adapt to MRSA, it is not surprising that the immune response against MRSA differs from that against well-conserved (non-MRSA) pathogens (as observed, here, in two species). Because vertebrates participate in both ‘old’ and ‘new’ interactions, there is no contradiction between the variability shown by individual birds and the similarity displayed by well-conserved functions, such as feedback.

Because MRSA pathogens, in addition to be resistant to anti-microbials, also induce immune failure, such pathogens may elicit abnormally high –although ineffective– N/L ratios [74]–[79], as found in bovines and humans. The highest values of such dysfunctional relationships were observed at the earliest observations (Figures 5 A–F). Because a high immune response can only be sustained for a limited time, such pattern could be used to distinguish early MRSA from late (MRSA and non-MRSA) responses.

### Methodological considerations

Methodological issues were also evaluated [80]. Because false negatives were documented, the hypothesis that there is an ideal test (‘gold standard’) was rejected [81], [82]. Because two or more D+ stages were distinguished, both the binary hypothesis (‘only two data classes’) and the assumption that all D+ data points have similar meaning were negated (Figures 1 and 6 B–E). One possible reason why those hypotheses were not empirically supported is that they do not account for dynamics and/or data circularity [83], [84].

Findings also addressed a problem, described as follows: in order to identify an infecting microbe, a specific test is needed; however, in order to choose such test, the identity of the pathogen should be known in advance. While the ‘gold standard’ could not solve this conundrum, the SB/EB approach provided an alternative for its solution [85].

### Consequences on theory

Findings may be used to rectify a concept previously espoused. Feedback loops do not differ in directionality, as suggested before [24]. The apparent change in directionality is an artifact due to earlier analyses, which did not consider 3D interactions: in 3D space, feedback loops reveal a single (circular) directionality.

Because feedback loops expressed temporal changes, causality was supported [24]. Thus, the 3D feedback-oriented analysis provided both descriptive and explanatory information.

Unlike approaches that dichotomize continuous data and generate D+ and D– data overlapping [62], the 3D analysis of feedback loops displayed data inflections, which resulted in minimal D+ and D– data overlapping. Such feature could be used to facilitate data partitioning.

Data structured to express feedback dynamics overcame the limitations of static approaches, as when Principal Component Analysis is used to assess compositional data [86]. Unlike prevalence – a static index [87]–, the proportion of subjects within early vs. late responses (information on dynamics) could distinguish populations with similar prevalence levels. Findings also showed that SB models can be applied across scales [88].

### Replications and applications

Across species, the SB/EB approach helped to recognize infectious disease data patterns. Because this study did not focus on the pathogenesis of any disease, the reproducibility of the findings should be investigated in future studies. Potential applications include: 1) early diagnosis, 2) error detection, 3) differentiation of D+ classes, 4) prognosis, 5) evaluation of interventions, and 6) modeling.

For instance, two or more D+ classes may be distinguished [89], [90]. High MC/N values (‘left overshooting’) could be used to predict recovery. When ‘right overshooting’ is observed (high N/L or P/L values) but no microbe is isolated, an infection cannot be ruled out (a false negative result may be suspected). To prevent delayed detection of MRSA cases [91], the SB/EB approach, which seemed to reveal early MRSA data patterns, could be considered.

The SB/EB approach may also be used to evaluate interventions and support modeling. For instance, the evaluation of interventions may distinguish the influence of feedback from the responsiveness of individuals: when an intervention seems to be a ‘success’, it could be asked whether such outcome is due to fast responders (a ‘false positive’ result), or, when a ‘failure’ appears to occur, whether it is due to slow responders (a ‘false negative’ result). In mathematical modeling, analyses that focus on MRSA-like infections could be optimized if the cyclic nature and directionality of feedback processes were addressed [92]–[94].

## Conclusions

More information related to infectious diseases can be extracted, using the same data, when some conditions are met. Findings document the influence of data structure on the amount and explanatory content of infectious disease-related information. Feedback-related patterns of 3D leukocyte structures may have broad applications, including earlier diagnosis and detection of errors.

## Supporting Information

Text S1 **Description on institutional approvals; descriptions on avian, bovine, and human studies; glossary; and data analysis.**
(DOC)
