# Supplementary material for: Feedback-Based, System-Level Properties of Vertebrate-Microbial Interactions
Source: PLoS One. 2013 Feb 20;8(2):e53984. doi: 10.1371/journal.pone.0053984 (PMC3577842; doi:10.1371/journal.pone.0053984)
Supplement: Text S1 — Description on institutional approvals; descriptions on avian, bovine, and human studies; glossary; and data analysis. (DOC) [file pone.0053984.s001.doc]

**SUPPORTING INFORMATION**

**Institutional approvals**

Prior to data collection, all studies, whether published or not, were approved by the appropriate ethics committees of the institutions here identified.

**Avian studies**

Specific pathogen free (SPF) white leghorn chicken embryos were obtained from Charles River Laboratories (Chicago, IL), hatched and raised until 6-weeks old at the University of Wisconsin-Madison, then transferred to BSL-3 facilities at the U.S. Geological Survey National Wildlife Health Center and acclimated for three weeks prior to infection experiments. Ten nine-week old chickens were randomized for subcutaneous injection with 100-µl bovine albumin-1 (BA-1) containing 10^5^ plaque-forming units of American crow isolate 16399-3 WNV and subsequently bled on days 1-5, 7, 10, and 14 DPI [52]. While not analyzed with the quantitative method here evaluated, the raw data here reported have been previously published [52]. The chickens utilized for this study were treated humanely with due consideration to the alleviation of their distress and discomfort, and according to University of Wisconsin-Madison Institutional Animal Care and Use Committee (IACUC) protocol #A01059 and US Geological Survey National Wildlife Health Center IACUC protocol #EP040811.

Laboratory techniques. Vero cells were used to detect the presence of virus in serum by plaque formation according to standard procedures [53]. Viremia (plaque forming units (PFU)/ml serum) was calculated from the serum dilution that produced between 5 and 30 plaques per well. Blood smears were created on the blood sampling days indicated above, treated with

Wright-Giemsa stain, and cell ratios were calculated once 100 WBCs were counted per slide via

light microscopy at 1000X. West Nile virus-infected chickens produced WNV- specific IgG and. especially IgM titers, were positively correlated with viremia. Uninfected chickens remained antibody and virus-negative (i.e., D–) throughout the duration of the study [52].

**Bovine studies**

Three studies were conducted with cows, from which microbial tests were conducted with milk samples: one longitudinal and experimental study (LE), one longitudinal study of an animal spontaneously infected with methicillin-resistant *S. aureus* (MRSA), and a cross-sectional study (CS) that included 6 populations located in 4 countries (CS I to VI, Table 3). In the LE study (conducted in the US), 6 lactating cows were inoculated intra-mammarily with *S. aureus*, and milk samples were investigated before and up to 14 days after challenge with *S. aureus* [44]. While not analyzed with the quantitative method here evaluated, the raw data here reported have been previously published [44].

In the MRSA study, conducted in Italy, all mammary glands of one infected cow were investigated at days 1-5, 8, and 9 (7 tests), where day 1 was the first day MRSA was isolated in milk cultures. While neither analyzed with the quantitative method here evaluated nor based on the samples here measured, the variables here assessed have been previously published [54].

Longitudinal comparisons between bovine MRSA and non-MRSA responses included study LE (non-MRSA, n=24) and the Italian (MRSA, n=28) study (total n=52).

All CS studies except CS II were conducted with non-periparturient cows. Table 3 describes the size, examples of bacterial species isolated, and the bacterial prevalence found in each CS study. CS I data are original. CS II-VI data, while not analyzed with the quantitative method here evaluated, have been partially or totally reported before [55-59], as described in the legend of Figure 4.

All bovines were treated according to protocols approved by the US Institutional and Animal Care and Use Committee or similar German, Israeli, and Italian agencies.

Laboratory techniques. The total milk leukocyte count/ml was quantified with a

Fossomatic 5000 (Hillerød, Denmark), DeLaval DCC (Tumba, Sweden), or a Bentley Somacount 150 (Bentley Instruments, Chaska, USA). Microbial cultures were performed in compliance with guidelines described elsewhere [60]. Ten μL(CS III and CS VI), 50 μL (MRSA, CS IV, and V), or 100 μL (CS I and II studies) of milk were cultured. Bovine leukocytes were identified and counted by cytology (all studies except CS III), flow cytometry (CS III), or both methods (LE study), as described elsewhere [44, 55-59]. Susceptibility patterns of bacterial isolates were determined using the disk diffusion method indicated below (see human studies).

**Human studies**

A - Malaria

Participants. Children aged 3-36 months (n=439) were recruited at Siaya District Hospital, Kenya, a holoendemic *P. falciparum* transmission area where residents may receive up to 300 infective mosquito bites per annum [61]. After the parent or guardian of the child provided written informed consent to participate in the study, a questionnaire was conducted to collect demographic and clinical information, including the signs and symptoms of the present illness. In order to minimize the effect of previous malarial infections and/or recent anti-malarial use, no child with either prior hospitalizations (for any reason) or treatment for malaria within

the previous two weeks was investigated. None of the recruited children had cerebral malaria,

non-falciparum malarial infections, bacteremia, HIV-1, or hookworm infections [62].

After enrollment, children were divided into three (two malaria-negative [M–] and one

malaria-positive [M+]) groups. Both M– groups were defined by absence of *P. falciparum* parasitemia. One M– subset was defined as neither infected, febrile, nor inflammed (NIFNI). The NIFNI subset acted as an internal control for the overall M– class. The M+ group was defined by presence of *P. falciparum* parasitemia (any density) on thin and thick peripheral blood smears. Children were then re-examined two weeks later for the presence of malaria parasitemia and hemoglobin concentrations. After the data were collected and based on data patterns, both the M– and the M+ data were divided into additional subsets, e.g., M– results not suspected or suspected to be false negative, and M+ results suspected or not suspected to be under recovery. Five children, suspected to be under recovery, were tested twice over two weeks, and their data included in some analyses, so the total number of observations, in such cases, was 444. Children were treated according to guidelines of the Ministry of Health, Kenya. The study was approved by the University of New Mexico and the Kenya Medical Research Institute.

B- Bacterial infections

Participants. Because bacteremia is highly prevalent among Kenyan children with malaria, 22 bacterial infections were evaluated in malaria-positive children. Based on the holoendemicity of malaria in this region, children with bacterial infections in the absence of malaria were not obtained. To control for the confounding effects of malaria, children with bacterial infections were matched according to age, gender, parasitemia, and hemoglobin concentrations. The subset of children co-infected with bacteremia and malaria included 8 multi-

drug resistant *S. aureus* (MRSA) and 14 non-MRSA isolates sensitive to, at least, oxacillin.

Laboratory techniques. No child received any intervention before samples were

collected. Asexual malaria trophozoites were determined as described before [62]. Thick and thin peripheral blood smears were prepared from venous blood samples and stained with Giemsa reagent for malaria parasite identification, and quantified by microscopy. Asexual malaria trophozoites were counted against 300 leukocytes based on absolute counts of white blood cells (WBC)/μL in whole blood. Parasite density was estimated as follows: parasites/μL = WBC count/μL x trophozoites/300. Complete blood counts were performed with a Beckman Coulter© Ac-T diff2™ (Beckman Coulter, Inc.).

Bacterial cultures were performed as previously described [63]. Briefly, blood cultures

were performed for all children upon enrollment into the study, and in suspected cases of bacteremia at acute febrile visits. Approximately, 1.0 mL of venipuncture blood was collected aseptically into sterile pediatric Isolator® microbial tubes (Wampole Laboratories, Princeton, USA) or directly inoculated into the pediatric blood culture bottle (Pediplus, Becton-Dickinson, Franklin Lakes, USA). Blood samples in the pediatric Isolator® microbial tubes were inoculated directly onto chocolate agar plates, while pediatric blood culture bottles were incubated in an automated BACTEC 9050 system (Becton-Dickinson) for 4 days. Positive cultures were examined by Gram stain and sub-cultured on blood agar, chocolate agar or MacConkey agar plates based on the Gram stain results.

Susceptibility patterns of the bacterial isolates were determined using the disk diffusion method according to the Clinical Laboratory Standards Institute guidelines [64]. Bacterial isolates were tested against disks of erythromycin (15 μg), trimethoprim-sulfamethoxazole (1.25/23.75 μg), nalidixic acid (30 μg), tetracycline (30 μg), ampicillin-salbactum (10/10 μg), ciprofloxacin (5 μg), chloramphenicol (30 μg), oxacillin (1 μg), amoxicillin-clavulanic acid (20/10 μg), doxycycline (30 μg), cefotaxime-clavulanic acid (30/10 μg) and gentamicin (10 μg). Methicillin-resistant *S. aureus* (MRSA) was detected using oxacillin disk followed by vancomycin testing. Control *S. aureus* (**ATCC** 25923 and 29213) strains were run concomitantly with the test organisms. For all of the microorganisms tested, resistance was

defined according to the Clinical Laboratory Standards Institute guidelines [64]. The study was approved by the Kenya Medical Research Institute.

**Glossary**

Three major constructs were assessed as defined:

1. Functional data integrity: a 2D/3D data structure that included at least two

variables which, together, included data from all cell types and described at least one biological function. This means that, collectively, the set (i) included data from L, M, and N, (ii) the data were structured in a way such that biological functions generated by multi-cellular interactions could be assessed (e.g., the L/M ratio), and (iii) higher-level biological functions (those that may result from interactions among interactions, such as the interplay between early and late responses could be measured (e.g., the simultaneous assessment of the N/L and M/N ratios).

1. Amplification: a data structure with numerical properties that enhance

pattern detection by virtue of measuring two ratios at the same time, in which the same variable is assessed twice (the numerator of one ratio is the denominator of the other ratio). For instance, the simultaneous assessment of the N/L and M/N ratios includes the same variable twice (the N%, in this example). Notice that ‘amplifier’ indicators do not necessarily possess ‘functional

data integrity’ (in this example, there is no L data).

1. Anchoring: a subset of functional data integrity in which two variables

suffice to produce a single line of observations, when one leukocyte ratio and one leukocyte

percentage are measured, in which the denominator of the ratio is the percentage being measured in another axis (e.g., the P/L vs. L %). Notice that ‘amplifier’ indicators, in 2D and, depending on the perspective considered, also in 3D plots, generate a single line of observations but do not necessarily produce amplification.

**Data analysis**

To generate a double-blind assessment, no researcher participated in all studies, and no

researcher involved in data collection participated in data analysis. Plots and descriptive

statistical tests were produced with *Minitab 15*, Minitab Inc., State College, PA, USA.
